# Reproductive toxicity of boron

**DOI:** 10.17179/excli2020-2757

**Published:** 2020-08-19

**Authors:** Florian Seidel

**Affiliations:** 1 Leibniz Research Centre for Working Environment and Human Factors, Ardeystr. 67, 44139 Dortmund, Germany

## ⁯⁯⁯⁯

***Dear Editor,***

Recently, Bolt and colleagues published a review article on possible effects of boron on human reproduction (Bolt et al., 2020[3]). The metalloid boron is required as an additive of fiberglass, structural material and ceramics. Human exposure typically occurs in mining. Since more than a decade, a discussion is ongoing concerning categorization of inorganic boron compounds as reproductive toxins (Moore, 1997[8]; Hasegawa et al., 2013[5]; ECHA, 2010[4]; SCCS, 2010[11]; ATSDR, 2010[1]). The authors summarized key findings relevant for this discussion (Bolt et al., 2020[3]):

- Boron causes testicular toxicity in rats, mice and dogs.
- The NOAEL in rats was 17.5 mg boron/kg body weight per day; at higher concentrations reduced epididymal sperm counts were observed.
- The NOAEL of developmental toxicity in rats was 9.6 mg boron/kg body weight per day; at higher doses fetal skeletal effects were observed.
- The boron blood concentrations in rats corresponding to the NOAEL of 9.6 mg/kg/day was in the range of 1,200 ng boron/g.
- Even for extreme occupational exposure groups in China and Turkey boron blood concentrations were clearly below 1,200 ng/g.

Evaluation of reproductive and developmental toxicity remains a major challenge (Sachinidis et al., 2019[10]; Leist et al., 2017[7]; Shinde et al., 2015[14], 2016[13], 2017[12]). Currently, several research projects aim at the development of *in vitro* tests of developmental toxicity (Waldmann et al., 2017[15]; Rempel et al., 2015[9]; Balmer et al., 2014[2]). For this purpose, human stem cells have been exposed to test compounds to study if their differentiation is compromised (Weng et al., 2014[17]; Waldmann et al., 2014[16]; Krug et al., 2013[6]). However, although mechanisms can be evaluated in these *in vitro* systems, it still remains challenging to extrapolate to the *in vivo* situation and derive e.g. NOAELs. Therefore, the evaluation of possible human developmental effects of boron still depends on animal experiments and as far as available on epidemiological studies. The systematic review of Bolt and colleagues suggests that occupational exposure of humans to boron results in boron blood concentrations that are clearly below levels that cause adverse effects in animal experiments. 

## Conflict of interest

The author declares no conflict of interest.
